# Surgical repair of a child with tetralogy of Fallot with absent left pulmonary artery: a case report

**DOI:** 10.1097/RC9.0000000000000007

**Published:** 2026-01-02

**Authors:** Alwaleed Al-Dairy, Ghaith Zain Aldien, Manar Ghanem, Ahmad Al-Bitar

**Affiliations:** a Cardiac Surgery at the Faculty of Medicine, Damascus University, Damascus, Syrian Arab Republic; b Faculty of Medicine, Damascus University, Damascus, Syrian Arab Republic

**Keywords:** absent left pulmonary artery (ALPA), congenital heart disease, postoperative hemoptysis, surgical complication, Tetralogy of Fallot (TOF)

## Abstract

**Introduction and importance:**

Tetralogy of Fallot (TOF) with unilateral absence of a pulmonary artery is a rare anomaly with challenging surgical management. Key risks include postoperative pulmonary hypertension due to a reduced pulmonary vascular bed. This report presents a case of TOF with absent left pulmonary artery (ALPA) that resulted in a fatal postoperative complication, highlighting a potentially underreported mechanism.

**Case presentation:**

A 5-year-old female with Tetralogy of Fallot with absent left pulmonary artery (TOF-ALPA) underwent successful surgical repair, including ventricular septal defect closure, right ventricular outflow tract augmentation, and patent ductus arteriosus (PDA) ligation with metallic clips. Her initial recovery was uneventful. However, on postoperative Day 4, she developed sudden massive hemoptysis and cardiac arrest, resulting in death despite prolonged resuscitation.

**Clinical discussion:**

The fatal hemoptysis was likely caused by a rupture of the solitary right pulmonary artery or a bronchovascular fistula. This is attributed to increased hemodynamic stress on the single artery post-repair and potential erosion from metallic PDA ligation clips. The event occurred despite the absence of pulmonary hypertension or coagulopathy, pointing towards structural or surgical factors.

**Conclusion:**

This case highlights the significant risk of lethal vascular complications in TOF-ALPA patients, even after technically successful surgery. It uniquely underscores the potential for catastrophic vascular erosion from metallic ligation clips in the context of a solitary pulmonary artery. This outcome calls for caution in the surgical approach, especially regarding materials used near fragile vasculature, and emphasizes the need for vigilant postoperative monitoring for delayed complications in this high-risk population.

## Introduction

Tetralogy of Fallot (TOF) with unilateral absence of pulmonary artery is a rare anatomical variation, occurring in 0.95%–3.23% of TOF cases^[1]^. Absence of the left pulmonary artery (ALPA) is more common, observed 5–8 times more frequently than right-sided absence^[2]^. In these patients, the affected lung receives blood supply from bronchial arteries or aortopulmonary collaterals, often due to premature closure of the ductus arteriosus during fetal development^[3]^.

Surgical management of Tetralogy of Fallot with absent left pulmonary artery (TOF-ALPA) is debated. Options include single-lung repair (relying on the contralateral pulmonary artery), double-lung repair, or palliative procedures^[2]^. Single-lung repair is considered if the contralateral pulmonary artery is normal-sized and the left ventricle is well-developed, ensuring adequate accommodation of right ventricular outflow (RVO)^[2]^. However, incomplete adaptation can lead to postoperative pulmonary hypertension (PH), requiring close follow-up to mitigate mortality risks^[2]^.

**HIGHLIGHTS**

- Surgical repair in patients with Tetralogy of Fallot with absent left pulmonary artery carries a high risk of fatal vascular complications due to inherent anatomical and hemodynamic vulnerabilities.
- A meticulous surgical approach, particularly regarding material choice near fragile vasculature, is essential to minimize the risk of postoperative hemorrhage and erosion.

Postoperative PH is a major concern in TOF-ALPA repair. A reduced pulmonary vascular bed capacity (≤50%) and elevated pulmonary-to-systemic resistance ratios (>0.85) contraindicate complete repair^[4,5]^. While some patients show excellent postoperative exercise tolerance^[4,6]^ others develop progressive pulmonary vascular disease, presenting as hemoptysis, cyanosis, or right heart failure^[6]^. Contributing factors include abnormal pulmonary vascular development in TOF^[6]^, post-repair pulmonary arterial vasospasm^[7,8]^, and insufficient contralateral lung capacity to handle RVO^[3]^.

While surgical repair is feasible, this case report describes the extremely rare and catastrophic complication of fatal hemoptysis on postoperative Day 4, highlighting critical gaps in our understanding of delayed vascular vulnerability and the choice of surgical materials in this specific anatomy. This report has been reported in line with the SCARE 2025 checklist^[9]^.

## Case presentation

**Figure 1.**
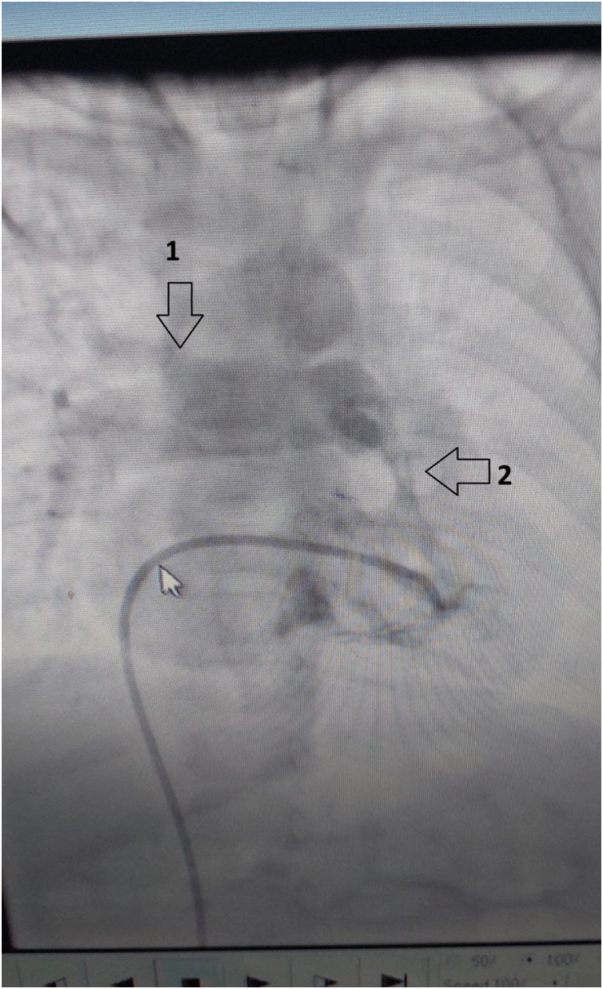
Preoperative cardiac catheterization image. Label “1” points to the solitary right pulmonary artery. Label “2” points to the right ventricular outflow tract. The absence of the left pulmonary artery is confirmed.

A 5-year-old Arab female (weight 18 kg, height 105 cm, body mass index (BMI) 16.3) was referred to our tertiary care institution for surgical repair of TOF-ALPA. She had no significant past medical history and no family history of congenital heart disease. Preoperative vital signs showed an oxygen saturation of 85% on room air, a heart rate of 130 bpm, and a blood pressure of 95/60 mmHg. Preoperative transthoracic echocardiography confirmed the diagnosis of TOF, demonstrating an absent left pulmonary artery (LPA), a large malaligned VSD with a peak gradient of 60 mmHg, preserved left ventricular (LV) size and function, and a normally sized right pulmonary artery (RPA). Cardiac catheterization further corroborated the anatomical findings, revealing a right ventricular pressure of 90 mmHg, systemic systolic pressure of 95 mmHg, and a well-developed RPA with no significant stenosis (Fig. 1). Following preoperative evaluation and medical optimization, elective surgical repair was scheduled.

**Figure 2.**
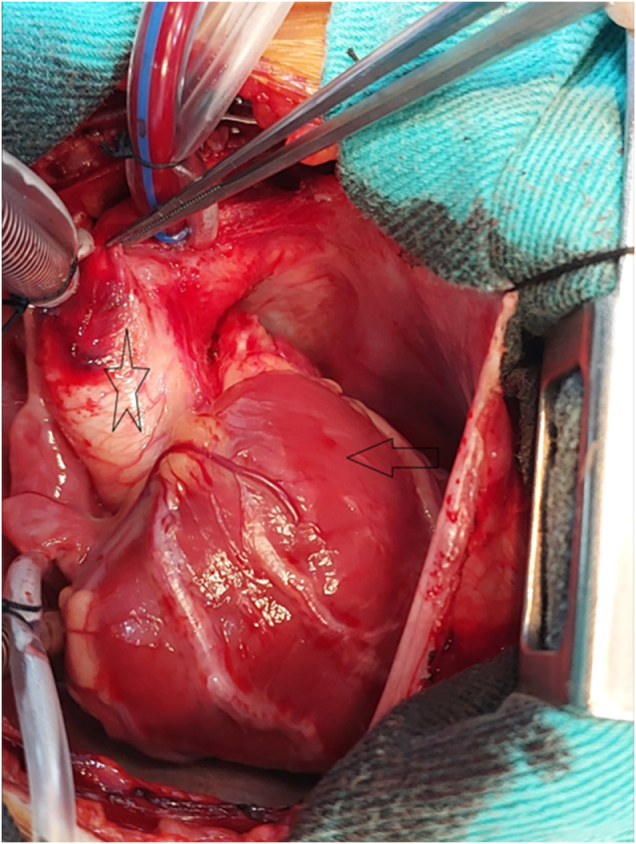
Intraoperative image after median sternotomy. The arrow points to the hypertrophied right ventricular outflow tract. The star indicates the ascending aorta. The expected location of the left pulmonary artery is notably absent.

**Figure 3.**
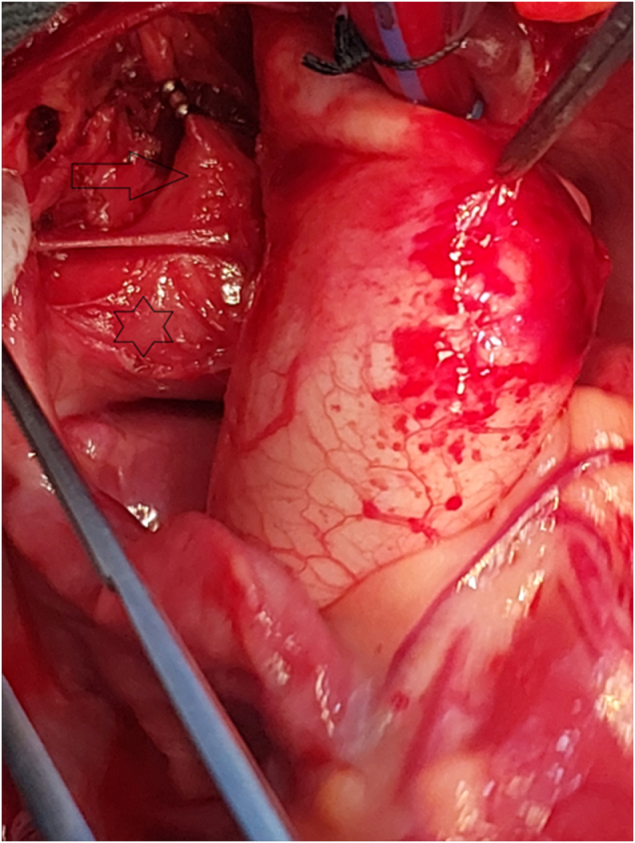
Intraoperative image focusing on the right pulmonary artery and ductus arteriosus. The star indicates the right pulmonary artery (RPA). The arrow points to the patent ductus arteriosus (PDA) after ligation with metallic clips.

The procedure was performed by a consultant congenital heart surgeon with over 15 years of experience in a center performing over 100 congenital heart surgeries annually. It was initiated via median sternotomy. Upon opening the pericardium, the absence of the LPA was visually confirmed (Fig. 2). Total cardiopulmonary bypass (CPB) was established using aortic and bicaval cannulation. The total CPB time was 120 minutes, and the aortic cross-clamp time was 85 minutes. Intraoperative exploration identified a large patent ductus arteriosus (PDA) supplying the RPA, which was ligated using metallic clips (Fig. 3). After aortic cross-clamping, antegrade cold blood cardioplegia was administered to achieve cardiac arrest.

Standard tetralogy repair was performed: the ventricular septal defect was closed through a right atrial approach with a Dacron patch, and the right ventricular outflow tract (RVOT) was addressed via resection of obstructive muscle bundles. The RVOT was subsequently augmented with a fresh autologous pericardial patch. The patient was successfully weaned from CPB without complications and transferred to the intensive care unit (ICU) on low-dose inotropic support (milrinone 0.5 mcg/kg/min and adrenaline 0.05 mcg/kg/min).

Her initial postoperative recovery in the ICU was uneventful. She was extubated on postoperative Day 1 and maintained stable hemodynamics (mean arterial pressure 65–75 mmHg, central venous pressure 10–12 mmHg). Anticoagulation with aspirin (5 mg/kg) was initiated. She was transferred to the general ward on postoperative Day 3 in stable condition. However, on postoperative Day 4, she developed progressive dyspnea and tachypnea, prompting urgent ICU readmission. Her clinical status rapidly deteriorated, necessitating reintubation. Shortly thereafter, she experienced massive hemoptysis with subsequent cardiac arrest. Despite 1 hour of advanced cardiopulmonary resuscitation, there was no return of spontaneous circulation, and the patient was declared deceased. Timeline of clinical events in Table 1.

**Table 1:** Timeline of clinical events

Timepoint	Event
Presentation	5-year-old female with known cyanotic heart disease referred for surgical repair.
Preoperative workup	TTE and cardiac catheterization confirm TOF with absent LPA and well-developed RPA.
Surgery (Day 0)	Successful complete repair: VSD closure, RVOT augmentation, PDA ligation with metallic clips. CPB time: 120 min, cross-clamp: 85 min.
Post-op Days 1–3	Uneventful recovery. Extubated on Day 1, transferred to ward on Day 3.
Post-op Day 4	Acute dyspnea, ICU readmission, reintubation, massive hemoptysis, cardiac arrest.
Outcome	Death despite 1 hour of resuscitation.

## Discussion

We present a case of fatal hemoptysis following successful TOF-ALPA repair, likely due to a combination of increased hemodynamic stress on a solitary RPA and erosion from a metallic PDA clip. This tragic outcome illustrates the precarious hemodynamic balance in such repairs.

The patient’s death likely stemmed from massive hemoptysis due to pulmonary artery rupture or bronchovascular fistula, directly linked to the unique anatomical vulnerabilities and surgical interventions. The ligation of the PDA with metallic clips introduced a clear risk of erosion into adjacent structures, such as the RPA or bronchial tree. This aligns with reported complications in TOF repairs, where foreign materials near delicate vascular structures can lead to late-onset fistulas or rupture^[1,9]^. For example, delayed erosion of clips has been documented in cases of ductal ligation, particularly in anatomically abnormal pulmonary vasculature^[1,9]^.

Furthermore, the absence of the LPA concentrated all pulmonary blood flow through the RPA. Post-repair, the augmented RVOT likely increased pressure and flow on the RPA, predisposing it to rupture. This is consistent with cases of TOF-ALPA, where unilateral pulmonary artery absence creates disproportionate hemodynamic strain on the remaining artery^[1,10]^. Studies highlight that such anatomical configurations elevate the risk of pulmonary artery aneurysms or tears^[11]^.

While the patient did not have absent pulmonary valve syndrome, which is associated with severe pulmonary artery dilation leading to compression or erosion of adjacent airways or vessels^[11]^, the absence of the LPA and ligation-related stress could mimic similar pathophysiology, leading to catastrophic bleeding^[11,12]^.

The delayed presentation (postoperative Day 4) aligns with subacute complications such as clip migration, pseudoaneurysm formation, or pressure-induced necrosis. Case reports describe similar timelines for vascular injuries post-TOF repair, particularly when foreign materials or altered hemodynamics are involved^[1,9]^. The absence of PH (common in TOF with pulmonary stenosis)^[12]^ and coagulopathy, as noted in the case, further narrows the cause to structural or surgical factors. Infective endocarditis or airway trauma from reintubation were less likely given the clinical context^[11]^.

## Strengths and limitations

A key strength of this report is the comprehensive preoperative and intraoperative documentation, providing a clear clinical timeline. The primary limitation is the absence of a post-mortem examination to definitively confirm the exact source of hemorrhage. As a single case report, it highlights a rare complication but cannot determine its incidence.

## Recommendations

This case offers three critical lessons for managing similar high-risk patients

1. Avoid metallic clips near the fragile pulmonary vasculature in single-lung perfusion scenarios; consider suture ligation or absorbable materials to minimize the risk of erosion.
2. Implement enhanced post-operative vigilance for delayed vascular complications, even in the absence of PH. A low threshold for cross-sectional imaging (e.g. computed tomography angiography) should be maintained if any respiratory or hemodynamic symptoms arise in the first postoperative week.
3. Preoperative planning for TOF-ALPA must account for the long-term hemodynamic burden on the solitary PA, potentially favoring RVOT reconstruction strategies that minimize late pressure stress on the arterial wall.

## Conclusion

This case highlights the precarious balance in managing TOF-ALPA, where even technically successful repair can be complicated by lethal postoperative vascular complications. The patient’s fatal hemoptysis likely resulted from hemodynamic stress on the solitary RPA and potential erosion from metallic PDA ligation clips, emphasizing anatomical vulnerabilities in unilateral pulmonary artery absence. The family expressed their profound grief and provided consent in the hope that sharing this outcome might contribute to improved safety for future patients. This report reinforces the need for individualized risk assessment, cautious material selection, vigilant postoperative monitoring, and long-term surveillance to optimize outcomes in this high-risk population.

## Data Availability

Not applicable.
